# Early- versus late-onset mortality trends in breast and female genital cancers in Spain, 1999–2024

**DOI:** 10.1007/s12094-026-04412-7

**Published:** 2026-06-03

**Authors:** Lucía Cayuela, Antoni Llueca, Julián Librero, Victoria Achaval, Francisco Herrero Machancoses, Aurelio Cayuela

**Affiliations:** 1 Department of Internal Medicine, Hospital Severo Ochoa, Leganés, Spain; 2 Department of Obstetrics and Gynecology, Reference Unit in Abdomino-Pelvic Oncological Surgery (UR-COAP) of Valencia Region, University General Hospital of Castellon, Castellón de La Plana, Spain; 3 Navarrabiomed-HUN-UPNA, Red de Investigación en Cronicidad, Atención Primaria y Prevención y Promoción de La Saluds (RICAPPS), Pamplona, Spain; 4 Department of Internal Medicine, Hospital de Móstoles, Móstoles, Spain; 5 Predepartmental Unit of Medicine, Faculty of Health Sciences, Jaume I University, 12071 Castellón de La Plana, Spain; 6 Independent Researcher, Seville, Spain

**Keywords:** Breast neoplasms, Genital neoplasms, Female, Mortality trends, Spain

## Abstract

**Background:**

Breast and gynaecological cancers represent the leading oncological burden among women worldwide. We aimed to describe long-term mortality trends for these malignancies in Spain over a 26 year period, focusing on differences between early-onset (< 50 years) and late-onset (≥ 50 years) disease.

**Methods:**

Mortality data (1999–2024) were obtained from the Spanish National Institute of Statistics. Age-standardised mortality rates (ASMRs) were calculated per 100,000 women using the 2013 European Standard Population. Joinpoint regression estimated annual percentage changes (APC) and average annual percentage changes (AAPC) with 95% confidence intervals (CI).

**Results:**

Total annual deaths rose from 9531 to 11,641, despite declining ASMRs for most sites. Breast cancer mortality decreased consistently (AAPC − 1.4%; 95% CI − 1.5, − 1.3), with a more pronounced reduction in the < 50 group (AAPC − 2.5%). Significant ASMR declines were observed for uterus NOS (− 2.3%), ovary (− 0.7%), and cervix uteri (− 0.6%). In the < 50 cohort, ovarian cancer mortality fell sharply (AAPC − 2.1%). Conversely, corpus uteri cancer was the only site showing an increasing trend (AAPC + 0.4%; 95% CI 0.1, 0.8), with a steeper increase observed among women ≥ 50 years. Non-monotonic trends were identified for less common gynaecological cancers.

**Conclusions:**

Mortality rates for breast and most gynaecological cancers in Spain have declined, although absolute deaths have increased. These trends are potentially consistent with advances in prevention and care alongside population ageing. The rise in corpus uteri cancer represents an emerging public health concern potentially associated with increasing metabolic risk factors, highlighting the need for targeted prevention strategies.

**Supplementary Information:**

The online version contains supplementary material available at https://doi.org/10.1007/s12094-026-04412-7.

## Introduction

Breast and gynaecological cancers, including malignancies of the breast, endometrium, ovary, and cervix, represent a major global health burden among women [1, 2]. In 2022, breast cancer alone accounted for 2.3 million new diagnoses and 666,000 deaths worldwide, remaining the most common cancer and leading cause of cancer mortality in women [3]. Gynaecological cancers contributed an additional 1.5 million cases and 680,000 deaths, with cervical cancer representing the largest proportion [2].

Within Europe, Spain consistently reports lower mortality from these cancers than most other countries [4–7]. Breast cancer mortality is the lowest in the European Union, with an age-standardised death rate of 12.8 per 100,000 women between 2001 and 2017, and a continuing downward trend [6]. After rising through the late twentieth century, mortality began to decline from the early 1990 s at about 2.1% per year, attributed to population-based screening, advances in systemic therapy, and behavioural shifts such as reduced hormone replacement therapy use [5, 8, 9].

In contrast, mortality trends across gynaecological cancers are more heterogeneous. Cervical cancer mortality declined by 3.2% annually between 1981 and 2003 but has since plateaued, reflecting reliance on opportunistic rather than organised screening [5, 7, 10, 11]. Ovarian cancer mortality increased until 1998 before stabilising, paralleling Western European patterns and improvements in oral contraceptive use, surgery, and chemotherapy [5, 12]. Uterine cancer mortality fell into the early 2000 s but has recently risen among older women [5], driven by the growing prevalence of obesity, a key factor in endometrial carcinogenesis [13].

Age-specific analyses reveal further complexity. Breast cancer mortality reductions are greatest among older women, consistent with screening effects, while younger women show differing aetiologies and higher rates of aggressive subtypes [5, 8]. Rising uterine cancer incidence in younger women [14] and recent increases in cervical cancer deaths under age 50 [15, 16] signal emerging public health challenges often masked by aggregate trends.

Despite comprehensive national data, long-term analyses of breast and major gynaecological cancers in Spain, particularly stratified by early (< 50 years) and late-onset (≥ 50 years) disease, remain limited. This study examines mortality trends from 1999 to 2024 using Joinpoint regression to clarify the contributions of screening, treatment, and risk factor changes, informing strategies for prevention, early detection, and clinical management.

## Methods

### Study design and setting

We conducted a nationwide, population‑based ecological study to examine long‑term mortality trends for breast and primary gynaecological cancers in Spain from 1999 to 2024. The starting year, 1999, was chosen to coincide with the full implementation of the International Classification of Diseases, 10th Revision (ICD‑10) in Spain’s national mortality registry, ensuring consistent cause‑of‑death classification. The study population comprised all women residing in Spain during this period, with complete national coverage provided through official mortality records.

### Data sources

Mortality data were obtained from the Instituto Nacional de Estadística (INE), which provides annual, standardised, and publicly accessible underlying cause‑of‑death records. The INE registry is highly reliable and ensures near‑complete ascertainment of national mortality. The primary outcome was mortality from breast and gynaecological malignancies classified according to ICD‑10. The following sites were included: breast (C50), cervix uteri (C53), corpus uteri and uterus, part unspecified (C54–C55), and ovary (C56). Other female genital cancers (C51–C52, C57–C58) were also recorded to provide a comprehensive overview of gynaecological mortality.

Census-level data were utilized as the population at risk for all registered deaths meeting these criteria, ensuring full population representation and eliminating sampling bias. Mortality was stratified by age group, with early‑onset disease defined as < 50 years and late‑onset disease as ≥ 50 years. These categories serve as proxies for premenopausal and postmenopausal status, respectively, reflecting established differences in tumour biology, hormonal risk factors, and exposure to screening programs [17].

### Statistical analysis

Temporal changes in mortality were quantified by calculating annual Crude Mortality Rates (CMR) and Age‑Standardised Mortality Rates (ASMR) per 100,000 women for each cancer site and age group. Age‑standardisation was performed using the direct method with the 2013 European Standard Population as the reference, enabling robust temporal and international comparisons.

Trends were modelled using Joinpoint regression, a validated method widely used in cancer epidemiology to identify statistically significant changes in temporal trend slopes (“joinpoints”) over time. The analysis was conducted using the Joinpoint Regression Program (Version 6.0.1, March 2026; National Cancer Institute, USA), applying the default settings. Given the 26 annual observations (1999–2024), the software automatically allowed a maximum of four joinpoints, in accordance with its built-in rule designed to prevent overfitting based on the number of data points. Model selection was performed using permutation tests as implemented in the program.

For each identified segment, Annual Percentage Changes (APC) and corresponding 95% confidence intervals (CI) were estimated. Average Annual Percentage Change (AAPC) was used to summarise overall trends across the entire study period. Analyses were conducted for all cancers overall and stratified by cancer type and age group to describe heterogeneity between early-onset and late-onset disease patterns. Statistical significance was defined as p < 0.05.

Given the descriptive nature of the study and the use of complete national data, no sensitivity analyses were performed. The objective was to characterise observed trends rather than assess causal relationships.

### Ethical considerations

As this study relied exclusively on anonymised, aggregated, and publicly accessible data, it did not require formal approval from an institutional ethics committee, in accordance with Spanish data protection regulations and the Declaration of Helsinki.

## Results

Between 1999 and 2024, ASMRs declined for most breast and gynaecological cancers in Spain (Table 1). Despite these favourable trends, the absolute number of deaths increased from 9531 to 11,641, potentially reflecting population growth and demographic ageing. Breast cancer (C50) remained the leading cause of cancer mortality. Its ASMR declined from 30.27 to 21.21 per 100,000 women (AAPC: − 1.4%), although the annual number of deaths rose by 15.5%.

**Table 1:** Age-standardised mortality rates (ASMR) and joinpoint regression analysis for female breast and genital cancers (all ages), Spain 1999–2024

Cancer site	ASMR 1999	ASMR 2024	AAPC (95% CI)	Period 1	APC (95% CI)	Period 2	APC (95% CI)
Breast (C50)	30.27	21.21	−1.4* (−1.5, −1.3)	1999–2024	−1.4* (−1.5, −1.3)		
Vulva (C51)	1.31	1.01	−0.8* (−1.1, −0.5)	1999–2024	−0.8* (−1.1, −0.5)		
Vagina (C52)	0.26	0.18	−1.5 (−2.8, 0.2)	1999–2006	−6.7* (−22.7, −0.4)	2006–2024	0.6 (−7.7, 11.1)
Cervix uteri (C53)	2.78	2.45	−0.6* (−0.9, −0.3)	1999–2024	−0.6* (−0.9, −0.3)		
Corpus uteri (C54)	3.93	4.31	0.4* (0.1, 0.8)	1999–2013	1.0* (0.6, 3.6)	2013–2024	−0.3 (−3.0, 0.3)
Uterus NOS (C55)	2.67	1.27	−2.3* (−2.6, −2.0)	1999–2024	−2.3* (−2.6, −2.0)		
Ovary (C56)	8.47	6.93	−0.7* (−0.9, −0.6)	1999–2024	−0.7* (−0.9, −0.6)		
Other female genital (C57)	0.89	0.69	−1.3* (−1.9, −0.7)	1999–2009	−4.3* (−8.3, −2.5)	2009–2024	0.7 (−0.2, 2.7)

*ASMR* age-standardised mortality rate, *AAPC*  average annual per cent change, *APC* annual per cent change, *CI* confidence interval. Rates expressed per 100,000 women, age-standardised using the 2013 European Standard Population. * Statistically significant (p < 0.05)

Among gynaecological malignancies, uterus, unspecified (C55), showed the greatest decline in mortality, with ASMR decreasing from 2.67 to 1.27 per 100,000 (AAPC: − 2.3%). In parallel, the number of deaths fell from 503 to 387 (− 23%). Ovarian (C56), cervical (C53), and vulvar cancers (C51) also showed declining ASMRs (AAPC range: − 0.6% to − 0.8%), although the absolute number of deaths increased, notably by 27% for ovarian cancer and 34% for cervical cancer. Corpus uteri cancer (C54) was the only site with increasing mortality (AAPC: + 0.4%), where deaths almost doubled (+ 78%). Joinpoint analysis identified an increasing trend between 1999 and 2013 (APC: + 1.0%), followed by stabilisation thereafter.

Less frequent cancers showed heterogeneous patterns. Vaginal cancer (C52) experienced a marked decline in ASMR between 1999 and 2006 (APC: − 6.7%) before stabilising, although the number of deaths increased slightly from 51 to 60 (+ 18%). Other and unspecified gynaecological cancers (C57) decreased until 2009 (APC: − 4.3%) and then plateaued.

Women aged < 50 years contributed relatively few deaths, and mortality rates remained low across all sites (Table 2). Nevertheless, this age group showed the steepest declines. Breast cancer mortality decreased by 2.5% annually (AAPC), with a 29% fall in absolute deaths. Ovarian cancer also declined substantially (AAPC: − 2.1%). Cervical cancer mortality followed a downward trend (AAPC: − 1.7%), while deaths remained stable. Uterus NOS (C55) ASMR also declined (AAPC: − 1.8%). By contrast, corpus uteri (C54) and vulvar cancer (C51) rates remained stable. It should be noted that due to the small number of events in this age group for sites like vulva and vagina, estimates may be subject to higher year-to-year variability.

**Table 2:** Age-standardised mortality rates (ASMR) and joinpoint regression analysis for female breast and genital cancers (< 50 years old), Spain 1999–2024

Cancer site	ASMR 1999	ASMR 2024	AAPC (95% CI)	Period 1	APC (95% CI)	Period 2	APC (95% CI)
Breast (C50)	6.41	3.42	−2.5* (−2.8, −2.2)	1999–2020	−2* (−2.2, −1.6)	2020–2024	−5* (−10.8, −2.6)
Vulva (C51)	0.02	0.04	0.5 (−1.9, 3.3)	1999–2024	0.5 (−1.9, 3.3)		
Vagina (C52)	0.02	0.01	non available				
Cervix uteri (C53)	1.04	0.81	−1.7* (−2.1, −1.2)	1999–2024	−1.7* (−2.1, −1.2)		
Corpus uteri (C54)	0.19	0.12	−0.3 (−1.6, 1.1)	1999–2024	−0.3 (−1.6, 1.1)		
Uterus NOS (C55)	0.2	0.16	−1.8* (−2.9, −0.7)	1999–2024	−1.8* (−2.9, −0.7)		
Ovary (C56)	1.35	0.75	−2.1* (−2.6, −1.6)	1999–2024	−2.1* (−2.6, −1.6)		
Other female genital (C57)	0.09	0.09	non available				

*ASMR* age-standardised mortality rate, *AAPC*  average annual per cent change, *APC* annual per cent change, *CI* confidence interval. Rates expressed per 100,000 women, age-standardised using the 2013 European Standard Population. *Statistically significant (p < 0.05)

Women aged ≥ 50 years accounted for the vast majority of deaths and consistently showed much higher ASMRs than younger women (Table 3). In this age group, breast cancer ASMR decreased (AAPC: − 1.3%), yet absolute deaths increased by 22%. A similar pattern of diverging rate and volume trends was observed for ovarian cancer (AAPC: − 0.6%; deaths: + 33%) and cervical cancer (AAPC: − 0.3%; deaths: + 45%).

**Table 3:** Age-standardised mortality rates (ASMR) and joinpoint regression analysis for female breast and genital cancers (50 + years old), Spain 1999–2024

Cancer site	ASMR 1999	ASMR 2024	AAPC (95% CI)	Period 1	APC (95% CI)	Period 2	APC (95% CI)
Breast (C50)	67.6	49.03	−1.3* (−1.4. −1.2)	1999–2024	−1.3* (−1.4. −1.2)		
Vulva (C51)	3.31	2.53	−0.8* (−1.1. −0.5)	1999–2024	−0.8* (−1.1. −0.5)		
Vagina (C52)	0.65	0.45	−1.4 (−2.8. 0.5)	1999–2006	−6.6 (−23.3, 0)	2006–2024	0.7 (−15, 12.1)
Cervix uteri (C53)	5.49	5.01	−0.3* (−0.6. −0.03)	1999–2024	−0.3* (−0.6. −0.03)		
Corpus uteri (C54)	9.78	10.86	0.5* (0.1. 0.8)	1999–2013	1* (0.6, 3.6)	2013–2024	−0.3 (−3, 0.3)
Uterus NOS (C55)	6.55	3.02	−2.3* (−2.7. −2)	1999–2024	−2.3* (−2.7. −2)		
Ovary (C56)	19.62	16.6	−0.6* (−0.7. −0.4)	1999–2024	−0.6* (−0.7. −0.4)		
Other female genital (C57)	2.13	1.64	−1.3* (−1.8. −0.8)	1999–2009	−4.3* (−7.3, −2.6)	2009–2024	0.7 (−0.2, 2.2)

*ASMR*  age-standardised mortality rate, *AAPC * average annual per cent change, *APC*  annual per cent change, *CI*  confidence interval. Rates expressed per 100,000 women, age-standardised using the 2013 European Standard Population. *Statistically significant (p < 0.05)

The most pronounced increase in absolute mortality occurred for corpus uteri cancer (C54), with deaths rising by 80% and ASMR increasing significantly (AAPC: + 0.5%). Joinpoint analysis confirmed an upward trend from 1999 to 2013 (APC: + 1.0%) followed by stabilisation. Vulvar cancer deaths increased by 34%, despite a modest decline in ASMR (AAPC: − 0.8%). In contrast, uterus NOS (C55) showed the clearest improvement, with deaths falling by 25% and a marked decline in ASMR (AAPC: − 2.3%). Vaginal cancer (C52) and other gynaecological cancers (C57) displayed declining or stable ASMRs, despite increases in absolute death counts.

## Discussion

This nationwide, population-based study shows that ASMRs for breast and most gynaecological cancers in Spain declined between 1999 and 2024, despite a 22.1% rise in absolute deaths.Query Corpus uteri cancer was the sole exception, showing a sustained increase in ASMRs, particularly among women aged ≥ 50 years. These results build on evidence of declining breast and uterine mortality since the early 1990 s [5], but reveal that uterine cancer trends have diverged by subsite. Spain maintains relatively low breast and cervical cancer mortality [18, 19], yet the growing burden of corpus uteri cancer, alongside rising colorectal and lung cancer mortality in women [20, 21], reflects an evolving epidemiological profile of cancer in women. These shifts are interpreted in light of clinical and demographic drivers, including screening impact, therapeutic innovation, and evolving population risk profiles.

The coexistence of declining ASMRs and rising absolute deaths represents an epidemiological paradox. While individual risk appears to have fallen, population ageing and an expanding pool of high-risk older women have driven the absolute burden upward. Consequently, healthcare demand in Spain continues to grow despite advances in prevention and treatment. This underscores the need to interpret mortality trends within a demographic-ageing framework, where longevity gains amplify absolute cancer burden despite improving individual prognosis.

Breast cancer mortality declined steadily (AAPC: − 1.4%), extending the downward trajectory initiated by mammographic screening and adjuvant therapies in the early 1990 s [5, 8]. This mirrors trends in high-income countries where therapeutic milestones, including anti-HER2 agents, CDK4/6 inhibitors, and immunotherapy, are associated with improved survival despite rising incidence [19]. This sustained decline may reflect a dominant period effect linked to the maturation of screening programs and the iterative integration of targeted therapies into routine Spanish clinical practice. Such progress may represent the translational success of transitioning molecularly-targeted interventions to population-level implementation.

Despite these clinical advances, the rate of decline in Spain remains below the World Health Organization (WHO) Global Breast Cancer Initiative target of a 2.5% annual reduction [22], suggesting that further progress could be achieved through better implementation of existing strategies or the adoption of new interventions. Furthermore, the absolute number of breast cancer deaths increased by 15.5% (from 5,684 to 6,563). This underscores a critical public health challenge: the benefits of therapeutic innovation may be progressively offset by demographic shifts. Specifically, the accelerated ageing of the population appears to have outweighed the impact of medical progress, leading to an increase in the total mortality burden even as individual prognosis improves.

Age-specific analyses showed marked heterogeneity. Among women aged ≥ 50 years, breast cancer ASMR declined modestly (AAPC: − 1.3%) while deaths increased by 22%, potentially associated with frailty, comorbidities, and delayed diagnosis. Furthermore, biological differences in late-onset tumours and less intensive treatment in older patients may limit mortality reductions. In contrast, women aged < 50 years saw a steeper decline (AAPC: − 2.5%), accelerating after 2020 (APC: − 5.0%). The wider use of anti-HER2 and CDK4/6 therapies, alongside multidisciplinary care, is a likely contributor to this trend [23, 24], suggesting that early-onset cancers may benefit disproportionately from aggressive therapeutic innovation.

Trends for gynaecological cancers were more heterogeneous. Cervical cancer mortality declined, though only modestly among women aged ≥ 50 years (AAPC: − 0.3%). This trend is consistent with the long-term impact of cytology-based screening and recent HPV vaccination [2, 10]. However, absolute deaths in older women rose by 45%, likely reflecting population ageing and gaps in historical screening coverage, a legacy of Spain’s traditionally opportunistic strategy [15]. This underscores how screening implementation may acts as a primary determinant of mortality trends, with younger, more consistently screened cohorts deriving greater benefit. Conversely, the sharper declines in women aged < 50 years likely reflect earlier diagnosis, therapeutic advances, and the initial population-level impact of HPV vaccination.

Mortality from vulvar cancer declined steadily throughout the study period (AAPC: − 0.8%), particularly among women aged ≥ 50 years, potentially reflecting earlier diagnosis of premalignant lesions, improved treatment of vulvar intraepithelial neoplasia, and growing recognition of HPV‑related vulvar cancers, although the absence of a significant decline in women aged < 50 years suggests that vaccination benefits have not yet fully emerged. Persistence of mortality in older women may be consistent with the increasing importance of non‑HPV‑related vulvar cancers associated with chronic inflammatory dermatoses such as lichen sclerosus [25].

Vaginal cancer remained the rarest major gynaecological malignancy, with ASMRs below 0.3 deaths per 100,000 women; mortality fell sharply until 2006 (APC: − 6.7%) but then stabilised, potentially due to improved detection and treatment of precursor lesions and the inherent instability of estimates for rare diseases. As many vaginal cancers are HPV‑related, wider HPV vaccination and organised cervical screening may yield further long‑term reductions, but progress has been limited in older women, who are less likely to have benefited from these programmes [26].

Ovarian cancer ASMR declined moderately, marking a significant departure from sharp pre-1998 increases [5, 12]. This progress likely reflects the long-term protective effects of oral contraceptives alongside therapeutic breakthroughs, including platinum-based chemotherapy and the introduction of PARP inhibitors [27]. Crucially, the centralization of care into specialized units may have improved survival for advanced cases by ensuring complete cytoreduction—the most significant prognostic factor for the disease [28]. These organizational improvements and maintenance therapies represent key period effects potentially driving mortality reductions. Despite these gains, absolute deaths rose by 27% overall, and 33% in women aged ≥ 50. Ovarian cancer remains highly lethal due to the prevalence of late-stage diagnosis, highlighting the need for earlier detection. and wider access to novel therapies.

In contrast, corpus uteri cancer was the only site with increasing ASMRs, with deaths rising by 78% overall and 80% among women aged ≥ 50 years. This reversal of the favourable pre-1990s trend [5] is consistent with international evidence linking endometrial cancer to obesity, metabolic syndrome, and type 2 diabetes [13, 14], factors that have become increasingly prevalent in Spain. In this context, these metabolic risk factors emerge as potential primary drivers of the observed mortality increase, reflecting a clear link between population health transitions and cancer outcomes. Furthermore, the trend may be influenced by a rising proportion of aggressive non-endometrioid tumours (e.g., serous and clear-cell carcinomas) and uterine sarcomas. Although 'uterus NOS' deaths declined by 23%, indicating improved certification, this sustained rise is unlikely to be attributed to coding shifts alone.

Overall, younger women appeared to benefit most from advances in prevention and treatment, showing marked mortality declines for breast, cervical, ovarian, and uterus NOS cancers [8]. Conversely, vulvar cancer mortality did not decline significantly in those aged < 50, and vaginal cancer remained too uncommon for clear trends. Older women continue to bear the greatest burden, particularly for cervical and corpus uteri cancers, highlighting persistent age-related inequalities. These age-specific disparities likely reflect a combination of divergent tumour biology (early- vs. late-onset disease) and differential uptake of screening and therapeutic innovations across generations, where younger cohorts benefit more from the translational shift toward personalized medicine.

These findings carry significant implications for cancer control. The sustained decline in breast and cervical cancer mortality supports continued investment in organized screening and modern therapies. However, Spain’s breast cancer ASMR reduction still falls short of the WHO 2.5% annual target, requiring better screening participation and minimized diagnostic delays. For ovarian cancer, priorities include expanding access to specialized centres and maintenance therapies (PARP inhibitors). The rising corpus uteri cancer burden suggests a need for a dual approach: primary prevention targeting the metabolic syndrome epidemic (obesity and sedentary behaviour) and secondary prevention through rapid referral pathways for early symptoms. For vulvar and vaginal cancers, high HPV vaccination coverage and the transition to organized HPV-based screening are essential. Ultimately, integrating population-level prevention with equitable access to innovative therapies is critical to overcoming the demographic challenge of an aging population and accelerating mortality reductions.

This study’s strengths include its nationwide design, 26 year longitudinal scope, and the use of Joinpoint regression to identify critical temporal inflection points. Stratification by age revealed mortality patterns that would otherwise have been obscured. While this approach is optimal for detecting shifts in trends, it does not fully disentangle age, period, and cohort effects. Limitations include the ecological design, which precludes causal inference. Additionally, the small number of events in women aged < 50 for certain sites may lead to less stable estimates. Although uterine cancer misclassification has decreased over time [5], changes in diagnostic criteria and ICD-10 coding may affect comparability. Future research utilizing Age–Period–Cohort models will further clarify the mechanistic drivers behind these observed trends.

In conclusion, mortality from breast and most gynaecological cancers in Spain has declined over the past 26 years, but the overall burden continues to rise due to population ageing. The increase in corpus uteri cancer deaths and the persistent excess among older women reveal important gaps in prevention and care. Addressing metabolic risk factors, optimizing screening programmes, and ensuring equitable access to therapeutic innovation will be critical to sustaining progress [29].

## Supplementary Information

Below is the link to the electronic supplementary material.

Supplementary file1 (DOCX 21 KB)

## Data Availability

The data supporting the conclusions of this study are publicly available at: https://www.ine.es/.

## References

[1] Zhang Y, Ji Y, Liu S, Li J, Wu J, Jin Q, et al. Global burden of female breast cancer: new estimates in 2022, temporal trend and future projections up to 2050 based on the latest release from GLOBOCAN. J Natl Cancer Cent. 2025;5:287–96. 10.1016/j.jncc.2025.02.002. PMC12276554 40693239

[2] Zhu B, Gu H, Mao Z, Beeraka NM, Zhao X, Anand MP, et al. Global burden of gynaecological cancers in 2022 and projections to 2050. J Glob Health. 2024;14:04155. 10.7189/jogh.14.04155. PMC11327849 39148469

[3] Gu H, Wang R, Beeraka NM, Lu P, Zhao X, Li L, et al. Global burden and trends of breast cancer: GLOBOCAN 2022 estimates of incidence and mortality in 185 countries. Chin Med J (Engl). 2026;139:404–14. 10.1097/CM9.0000000000003921. PMC12875702 41473983

[4] Bosetti C, Bertuccio P, Malvezzi M, Levi F, Chatenoud L, Negri E, et al. Cancer mortality in Europe, and an overview of trends since 1980. Ann Oncol. 2013;24:2657–71. 10.1093/annonc/mdt301. 23921790

[5] Cabanes A, Vidal E, Pérez-Gómez B, Aragonés N, López-Abente G, Pollán M. Age-specific breast, uterine and ovarian cancer mortality trends in Spain: changes from 1980 to 2006. Cancer Epidemiol. 2009;33:169–75. 10.1016/j.canep.2009.08.010. 19766076

[6] Jani C, Salcicciol I, Rupal A, Al Omari O, Goodall R, Salciccioli JD, et al. Trends in breast cancer mortality between 2001 and 2017: an observational study in the European Union and the United Kingdom. JCO Glob Oncol. 2021;7:1682–93. 10.1200/GO.21.00288. PMC8691519 34910553

[7] Salciccioli I, Zhou CD, Okonji EC, Shalhoub J, Salciccioli JD, Marshall D. European trends in cervical cancer mortality in relation to national screening programs, 1985-2014. Cancer Epidemiol. 2021;74:102002. 10.1016/j.canep.2021.102002. 34371420

[8] Sanvisens A, Vidal-Vila A, Puigdemont M, Viñas G, Roqué-Lloveras A, Del Barco S, et al. Population-based analysis of breast cancer incidence and mortality: overall and age-specific temporal trends over 40-year period in Girona, Spain. Breast Cancer Res Treat. 2025;212:97–105. 10.1007/s10549-025-07704-8. PMC12086112 40281345

[9] Vidal Lancis C, Martínez-Sánchez JM, Mateos Mazón M, Peris Tuser M. [Breast cancer mortality trend in Spain and its autonomous communities during the period 1980-2005]. Rev Esp Salud Publica. 2010;84:53–9. 10.1590/s1135-57272010000100006. 20376413

[10] Cervantes-Amat M, López-Abente G, Aragonés N, Pollán M, Pastor-Barriuso R, Pérez-Gómez B. The end of the decline in cervical cancer mortality in Spain: trends across the period 1981-2012. BMC Cancer. 2015;15:287. 10.1186/s12885-015-1306-x. PMC4408572 25886170

[11] Rodríguez-Rieiro C, Aragonés N, Pollan M, López-Abente G, Pérez-Gómez B. [Temporal trends of cervical cancer mortality rates among regions in Spain: 1975-2004]. Med Clin (Barc). 2009;133:41–6. 10.1016/j.medcli.2008.11.034. 19457499

[12] Dalmartello M, La Vecchia C, Bertuccio P, Boffetta P, Levi F, Negri E, et al. European cancer mortality predictions for the year 2022 with focus on ovarian cancer. Ann Oncol. 2022;33:330–9. 10.1016/j.annonc.2021.12.007. 35090748

[13] Frias-Gomez J, Peremiquel-Trillas P, Alemany L, Ameijide A, Marcos-Gragera R, Ponce J, et al. Predicting the rising incidence and mortality of endometrial cancers among women aged 65-74 years in Catalonia. Maturitas. 2021;144:11–5. 10.1016/j.maturitas.2020.09.006. 33358202

[14] Petrova D, Sánchez-Martín A, Redondo-Sánchez D, Madrid Pérez-Esparza B, Suárez Betancourt L, González-Flores E, et al. What cancers are increasing in young adults: a population-based study of incidence trends in adults 20-49 years old in the South of Spain. BMC Public Health. 2025;26:16. 10.1186/s12889-025-25707-4. PMC12764024 41275217

[15] Dabán-López P, Fernández-Martínez NF, Petrova D, Rodríguez-Barranco M, Jiménez-Moleón JJ, Gutierrez J, et al. Epidemiology of human papillomavirus-associated anogenital cancers in Granada: a three-decade population-based study. Front Public Health. 2023;11:1205170. 10.3389/fpubh.2023.1205170. PMC10537955 37780447

[16] Llorca J, Rodriguez-Cundin P, Dierssen-Sotos T, Prieto-Salceda D. Cervical cancer mortality is increasing in Spanish women younger than 50. Cancer Lett. 2006;240:36–40. 10.1016/j.canlet.2005.08.021. 16229942

[17] Rantala J, Seppä K, Eriksson J, Heinävaara S, Härkänen T, Jousilahti P, et al. Incidence trends of early-onset breast cancer by lifestyle risk factors. BMC Cancer. 2025;25:326. 10.1186/s12885-025-13730-y. PMC11846213 39984883

[18] Santucci C, Mignozzi S, Levi F, Malvezzi M, Boffetta P, Negri E, et al. European cancer mortality predictions for the year 2025 with focus on breast cancer. Ann Oncol. 2025;36:460–8. 10.1016/j.annonc.2025.01.014. 40074664

[19] Zhong H, Yu S, Huang Y, Xuan F. Temporal trends in mortality of female cancers among women and the impact of COVID-19 in low- and middle-income countries, 1990-2023: an age-period-cohort analysis from the Global Burden of Disease Study 2023. Int J Womens Health. 2026;18:581359. 10.2147/IJWH.S581359. PMC12927747 41736760

[20] Cayuela L, Achaval V, Flox-Benítez G, Cayuela Domínguez A. Trends in early- and late-onset gastrointestinal cancer mortality in Spain, 1999–2023. Rev Esp Enferm Dig. 2025;117(10):564–71. 10.17235/reed.2025.11389/2025. 40575889

[21] Cayuela L, Gaeta AM, Achaval Rodríguez V, Annunziata A, Fiorentino G, Cayuela A. Declining early-onset lung cancer mortality in Spain: sex-specific trends and regional variations, 1999-2022. Cancer Epidemiol. 2025;97:102816. 10.1016/j.canep.2025.102816. 40209458

[22] Kim J, Harper A, McCormack V, Sung H, Houssami N, Morgan E, et al. Global patterns and trends in breast cancer incidence and mortality across 185 countries. Nat Med. 2025;31:1154–62. 10.1038/s41591-025-03502-3. 39994475

[23] Berton Giachetti PPM, Morganti S, Gandini S, Giudici F, Marra A, Nicolò E, et al. Survival following CDK4/6 inhibitor therapy for hormone receptor-positive, ERBB2-negative metastatic breast cancer. JAMA Netw Open. 2025;8:e2461067. 10.1001/jamanetworkopen.2024.61067. PMC11846014 39982725

[24] Ismaili N. A systematic review of major advances in breast cancer therapeutics in 2025: synthesis of conference and published evidence. Int J Mol Sci. 2026;27:1971. 10.3390/ijms27041971. PMC12941040 41752108

[25] Vieira-Baptista P, Pérez-López FR, López-Baena MT, Stockdale CK, Preti M, Bornstein J. Risk of development of vulvar cancer in women with lichen sclerosus or lichen planus: a systematic review. J Low Genit Tract Dis. 2022;26:250–7. 10.1097/LGT.0000000000000673. 35285455

[26] Montero-Macías R, Adam M, Rouzier R, Bonneau C. HPV vaccination efficacy in primary and tertiary prevention of vulvar and vaginal HPV-related high grade dysplasia and cancers: a systematic review. Hum Vaccin Immunother. 2025;21:2567704. 10.1080/21645515.2025.2567704. PMC12574581 41158101

[27] Bray F, Loos AH, Tognazzo S, La Vecchia C. Ovarian cancer in Europe: cross-sectional trends in incidence and mortality in 28 countries, 1953-2000. Int J Cancer. 2005;113:977–90. 10.1002/ijc.20649. 15505879

[28] Llueca A, Serra A, Climent MT, Segarra B, Maazouzi Y, Soriano M, et al. Outcome quality standards in advanced ovarian cancer surgery. World J Surg Oncol. 2020;18:309. 10.1186/s12957-020-02064-7. PMC7690155 33239057

[29] Llueca A, Canete-Mota S, Jaureguí A, Barneo M, Ibañez MV, Neef A, et al. The impact of liquid biopsy in advanced ovarian cancer care. Diagnostics (Basel). 2024;14:1868. 10.3390/diagnostics14171868. (**Basel, Switzerland**). PMC11394565 39272653

