# Supplementary material for: Early- versus late-onset mortality trends in breast and female genital cancers in Spain, 1999–2024
Source: Clin Transl Oncol. 2026 Jun 3;28(11):5213–20. doi: 10.1007/s12094-026-04412-7 (PMC13694481; doi:10.1007/s12094-026-04412-7)
Supplement: Supplementary file 1 [file 12094_2026_4412_MOESM1_ESM.docx]

# STROBE Checklist

*Early- versus Late-Onset Mortality Trends in Breast and Female Genital Cancers in Spain, 1999–2024*

Study design: Nationwide population-based ecological study

| **Item** | **Recommendation** | **Status** | **Where addressed in manuscript** |
| --- | --- | --- | --- |
| **TITLE & ABSTRACT** | | | |
| **1a** | Indicate the study design in the title or abstract | **✅** | Title includes "Mortality Trends"; Methods describe a "nationwide, population-based ecological study" using Joinpoint regression |
| **1b** | Provide an informative abstract | **✅** | Abstract summarises background, methods, key findings, and conclusions |
| **INTRODUCTION** | | | |
| **2** | Explain scientific background and rationale | **✅** | Introduction, paragraphs 1–5 |
| **3** | State specific objectives | **✅** | Final paragraph of Introduction: to examine mortality trends in Spain, 1999–2024, stratified by age group using Joinpoint regression |
| **METHODS** | | | |
| **4** | Present key elements of study design early | **✅** | Methods, "Study design and setting" |
| **5** | Describe setting, locations, and dates | **✅** | Methods, "Study design and setting" and "Data sources": Spain, 1999–2024, ICD-10 national mortality registry |
| **6a** | Describe eligibility criteria and data sources | **✅** | Methods, "Study design and setting": all women residing in Spain with deaths registered under selected ICD-10 codes |
| **6b** | Matching criteria (if applicable) | **N/A** | Not applicable to this study design |
| **7** | Define outcomes, exposures, and potential confounders | **✅** | Methods, "Data sources": outcomes defined by ICD-10 codes C50–C58; age groups <50 and ≥50 years as proxy for menopausal status; confounders not applicable in this descriptive ecological design |
| **8** | Describe data sources and measurement methods | **✅** | Methods, "Data sources": mortality data from the Instituto Nacional de Estadística (INE); ASMRs calculated using the 2013 European Standard Population |
| **9** | Describe efforts to address bias | **✅** | Discussion, limitations paragraph: acknowledges ecological design limitations, possible misclassification of uterine cancer deaths (C53–C55), and potential impact of ICD-10 coding changes over time |
| **10** | Explain study size | **✅** | Methods, "Data sources": census-level inclusion of all registered deaths in Spain; no sampling or power calculation required |
| **11** | Explain handling of quantitative variables | **✅** | Methods, "Statistical analysis": mortality expressed as ASMR per 100,000 women; age dichotomised as <50 years (early-onset) and ≥50 years (late-onset) |
| **12a** | Describe statistical methods | **✅** | Methods, "Statistical analysis": Joinpoint regression; APC and AAPC with 95% CI; significance threshold p<0.05; Joinpoint Regression Program v6.0.1 (NCI) cited |
| **12b** | Describe subgroup analyses | **✅** | Methods and Results: analyses stratified by cancer site and age group (<50 vs ≥50 years) |
| **12c** | Explain handling of missing data | **✅** | Not applicable; INE national mortality registry data are complete and aggregated at census level |
| **12d** | Explain loss to follow-up or sampling strategy | **N/A** | Not applicable; complete national registry with no follow-up or sampling |
| **12e** | Describe sensitivity analyses | **N/A** | The study utilized a complete national census (INE data) rather than a sample, eliminating sampling error. Furthermore, the study's aim was descriptive/trend characterization (identifying what happened) rather than causal inference (identifying why it happened at an individual level), rendering sensitivity analyses for confounding variables outside the primary scope. |
| **RESULTS** | | | |
| **13a** | Report numbers at each stage | **✅** | Results: total deaths in 1999 and 2024 reported overall and by cancer site |
| **13b** | Give reasons for non-participation | **N/A** | Not applicable; complete national registry |
| **13c** | Consider a flow diagram | **N/A** | Not necessary given use of national registry data |
| **14a** | Give characteristics of study participants | **✅** | Results: age-stratified analyses presented for all cancer sites (<50 vs ≥50 years) |
| **14b** | Report missing data | **N/A** | No missing data; complete registry |
| **14c** | Summarise follow-up time | **N/A** | Not applicable to this ecological design |
| **15** | Report outcome events or summary measures | **✅** | Results tables and text: ASMRs, APCs, AAPCs, and absolute deaths reported for each cancer site and age group |
| **16a** | Provide estimates and precision | **✅** | Results tables include AAPCs with 95% confidence intervals for all cancer sites |
| **16b** | Report category boundaries | **✅** | Methods: early-onset defined as <50 years; late-onset as ≥50 years |
| **16c** | Translate relative into absolute risk where relevant | **✅** | Results provide both ASMRs (relative measure) and absolute numbers of deaths |
| **17** | Report other analyses | **✅** | Results include subgroup analyses by age group and cancer site; Joinpoint segments and trend periods described for each site |
| **DISCUSSION** | | | |
| **18** | Summarise key results | **✅** | Discussion, first paragraph |
| **19** | Discuss limitations | **✅** | Discussion, penultimate paragraph: ecological design, absence of individual-level data, possible misclassification of uterine cancer deaths, ICD coding changes, and use of age 50 as menopausal proxy |
| **20** | Provide cautious overall interpretation | **✅** | Discussion and Conclusion: findings interpreted in the context of existing evidence with appropriate caveats |
| **21** | Discuss generalisability (external validity) | **✅** | Discussion: findings contextualised within Spain and compared with other high-income and European countries |
| **OTHER** | | | |
| **22** | State source of funding | **✅** | "No external funding was received for this study |

*Status legend: ✅ Addressed	N/A Not applicable*
